# Comparison of an Oscillometric Method with Cardiac Magnetic Resonance for the Analysis of Aortic Pulse Wave Velocity

**DOI:** 10.1371/journal.pone.0116862

**Published:** 2015-01-22

**Authors:** Hans-Josef Feistritzer, Sebastian J. Reinstadler, Gert Klug, Christian Kremser, Benjamin Seidner, Regina Esterhammer, Michael F. Schocke, Wolfgang-Michael Franz, Bernhard Metzler

**Affiliations:** 1 University Clinic of Internal Medicine III, Cardiology and Angiology, Medical University of Innsbruck, Innsbruck, Austria; 2 Department of Radiology I, Medical University of Innsbruck, Innsbruck, Austria; Northwestern University Feinberg School of Medicine, UNITED STATES

## Abstract

**Objectives:**

Pulse wave velocity (PWV) is the proposed gold-standard for the assessment of aortic elastic properties. The aim of this study was to compare aortic PWV determined by a recently developed oscillometric device with cardiac magnetic resonance imaging (CMR).

**Methods:**

PWV was assessed in 40 volunteers with two different methods. The oscillometric method (PWV_OSC_) is based on a transfer function from the brachial pressure waves determined by oscillometric blood pressure measurements with a common cuff (Mobil-O-Graph, I.E.M. Stolberg, Germany). CMR was used to determine aortic PWV_CMR_ with the use of the transit time method based on phase-contrast imaging at the level of the ascending and abdominal aorta on a clinical 1.5 Tesla scanner (Siemens, Erlangen, Germany).

**Results:**

The median age of the study population was 34 years (IQR: 24–55 years, 11 females). A very strong correlation was found between PWVOSC and PWVCMR (r = 0.859, p < 0.001). Mean PWV_OSC_ was 6.7 ± 1.8 m/s and mean PWVCMR was 6.1 ± 1.8 m/s (p < 0.001). Analysis of agreement between the two measurements using Bland-Altman method showed a bias of 0.57 m/s (upper and lower limit of agreement: 2.49 m/s and -1.34 m/s). The corresponding coefficient of variation between both measurements was 15%.

**Conclusion:**

Aortic pulse wave velocity assessed by transformation of the brachial pressure waveform showed an acceptable agreement with the CMR-derived transit time method.

## Introduction

Since aortic stiffness has emerged as an independent predictor of cardiovascular morbidity and mortality [1–3], non-invasive estimation of aortic elastic properties is highly desirable. Measurement of aortic pulse wave velocity (PWV) is the gold standard for the assessment of aortic stiffness [2]. It is defined as the propagation velocity of the pulse wave and is inversely correlated with vascular elasticity.

Phase-contrast cardiovascular magnetic resonance (CMR) provides an accurate, non-invasive method to determine various parameters of aortic stiffness [4–6]. For instance, the transit time method is a valid and highly reproducible technique for the assessment of aortic PWV and shows good agreement with invasive catheter measurements [7]. Recently, a new oscillometric method has been developed that calculates aortic PWV by means of transformation of the brachial pressure waveform [8, 9]. First validation studies suggest a very strong correlation with invasive intra-aortic catheter measurements [10].

Aortic PWV has recently been assessed by CMR in patients after acute ST-segment elevation myocardial infarction [11]. Furthermore, increased PWV has been linked to biomarkers of elevated myocardial wall stress in these patients [12]. Nevertheless, CMR as used in this study is an expensive imaging modality that is still not widely available, with application restricted to clinically stable patients [13]. Therefore, a simple and widely available tool that can be used also for assessing unstable, critically ill patients is highly desirable.

Transformation of the brachial pressure waveform using a recently developed oscillometric device provides a new method for assessing aortic PWV. So far, this approach has not been compared with the CMR-derived transit time method. The aim of the present study was to compare the new method with velocity-encoded phase-contrast CMR for the assessment of aortic PWV.

## Materials and Methods

### Study Population

Forty volunteers were enrolled in this prospectively designed validation study. They were all screened with a standardized questionnaire. All subjects were free from any symptoms attributable to acute and chronic cardiovascular disease. The study was approved by the ethics committee of Innsbruck Medical University. Written informed consent was obtained from all subjects before study inclusion.

Oscillometric measurements were performed once within 15 minutes after CMR scans.

### Oscillometric pulse wave analysis

Brachial pulse wave analysis was performed using Mobil-O-Graph (I.E.M., Stolberg, Germany). This device is a commercially available brachial oscillometric ambulatory blood pressure monitor and has been validated according to European Society of Hypertension recommendations [14]. A common cuff was centered to the left upper arm. Cuff size was chosen according to the circumference of the mid upper arm.

**Figure 1:**
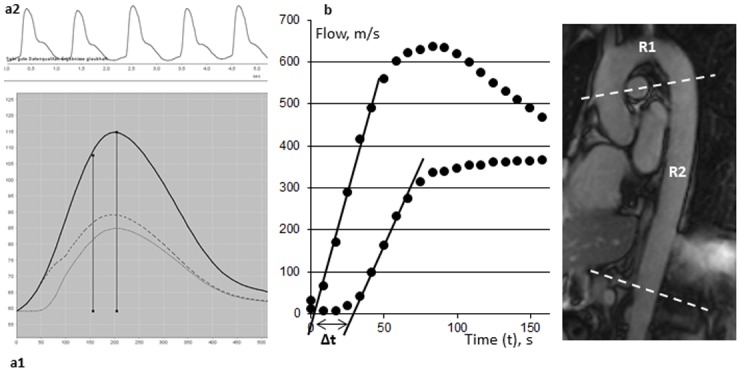
Assessment of aortic pulse wave velocity. Evaluation of aortic pulse wave velocity using (a1) a transfer function from (a2) brachial pressure wave analysis and (b) the cardiac magnetic resonance-derived transit time method based on phase-contrast imaging. R1 and R2 indicate the aortic region.

Generation of central aortic blood pressure curves based on brachial pulse waves is based on a previously published algorithm which integrates arterial impedance and aortic hemodynamics into a mathematical model [9, 15]. At first, brachial pressure wave forms are tested for plausibility and screened for artefacts (Fig. 1a). Thereafter, the ARCSolver method allows for PWV calculation using data derived from pulse wave analysis and wave separation analysis. These data, together with aortic characteristic impedance, age, sex are transformed using a previously described mathematical model for PWV_OSC_ assessment [9, 10, 16].

### Cardiac magnetic resonance

All scans were performed on a 1.5 Tesla Magnetom AVANTO-scanner (Siemens, Erlangen, Germany). The velocity-encoded phase-contrast sequence used in this study is a component of our generally used CMR protocol [17, 18]. Velocity encoding was set to 150 cm/s and was adjusted in the case of aliasing. Spatial resolution was 1.33 mm x 1.33 mm x 8 mm. Repetition time (TR) was 13.56 ms. Retrospective ECG triggering with 128 phases per cardiac cycle was applied. The mean heart rate during CMR scans was 66 ± 11 beats per minute. Consequently, the reconstructed mean temporal resolution was 7.1 ms.

### Determination of aortic stiffness using CMR

To plan optimal position of acquisition levels, an oblique sagittal slice was fitted to the aorta (Fig. 1b). Acquisition planes were set perpendicular to the ascending aorta, transecting both the ascending and descending thoracic aorta and perpendicular to the abdominal aorta below the diaphragm as described previously [12, 19]. Image evaluation was performed using standard software (ARGUS, Siemens, Erlangen, Germany). Aortic contours were circled manually and through-plane flow (ml/s) was calculated using the velocity values of the velocity-encoded images.

CMR-derived aortic PWV (PWV_CMR_) was defined as the mean propagation velocity between the ascending and abdominal aorta assessed by the classical transit time method. Accordingly, PWV was calculated as the ratio of the distance between aortic levels (Δx) and the propagation time of the pulse wave (Δt) between these sites.

$$PWV_{CMR}=\Delta x/\Delta t$$ (1)

The systolic upstroke of the flow curve reflected the arrival of the pulse wave at the level of measurement. A regression line was automatically fitted to the linear phase of the systolic upslope. The gap between the intersections, where the regression lines cross the x-axis, was defined as the travel time of the pulse wave between the two sites of measurement. Distance between these sites was measured manually along the aortic luminal midline on the oblique sagittal slice.

Aortic distensibility coefficients (DC) were assessed at the level of the ascending, descending thoracic and abdominal aorta. All 128 phases were screened for maximum and minimum aortic cross-sectional area. Contours were drawn manually on corresponding magnitude images. Peripheral as well as central pulse pressure was assessed subsequently to CMR imaging using the oscillometric device. Local aortic DC was then calculated as the ratio of the relative lumen change (ΔA/A) during systole and the pulse pressure (ΔP).

DC=ΔA/(A×ΔP) (2)

Our study group has proven excellent inter- and intraobserver variability for PWV_CMR_ and DC assessment [19].

### Statistical analysis

All analyses were performed using SPSS 19.0.0 (IBM, Armonk, NY, USA). Kolmogorov-Smirnov test was applied to test for normal distribution. All parameters except age of the overall study population were normally distributed. All the results are expressed as mean ± standard deviation or as median with interquartile range (IQR) if not normally distributed. Pearson`s test or Spearman`s rank correlation coefficients were calculated according to the distribution of variables. Differences in continuous variables between groups were assessed by t-test or Mann-Whitney U test. Fisher´s r-to-z transformation was applied for comparison of correlation coefficients. Bland-Altman plots were created to analyse the agreement between the methods. For the assessment of the coefficients of variation, the standard deviation of the differences between the two methods was divided by the mean value of both methods. Two-tailed p values < 0.05 were considered to indicate statistical significance.

## Results

### Study population

Baseline characteristics of the study cohort are summarized in table 1. The median age of the study population was 34 years (IQR: 24–55 years) and ranged between 19 and 71 years. The difference in age between males and females was not statistically significant (42 ± 18 years vs. 33 ± 12 years, p = 0.148). Hypertension and hyperlipidemia were present in 5 (13%) and 4 (10%) subjects. Furthermore, 4 (10%) subjects were current smokers.

**Table 1:** Study population characteristics.

	**Study population (n = 40)**
Age, years	34 [24–55]
Female, n (%)	11 (28)
Body mass, kg	74 ± 12
Height, cm	178 ± 9
Body mass index, kg/m^2^	23.4 ± 2.4
Body surface area, m²	1.9 ± 0.2
RR_sys_, mmHg	129 ± 14
RR_dia_, mmHg	85 ± 11

RR_sys_: systolic blood pressure, RR_dia_: diastolic blood pressure.

Oscillometric as well as CMR-derived parameters of aortic stiffness are summarized in table 2. PWV_OSC_ and PWV_CMR_ ranged between 4.5 m/s and 10.4 m/s as well as 3.9 m/s and 10.9 m/s. PWV_OSC_ (7.0 ± 1.9 m/s vs. 5.6 ± 1.1 m/s, p = 0.006) and PWV_CMR_ (6.4 ± 2.0 m/s vs. 5.2 ± 0.5 m/s, p = 0.008) was higher in males than in females.

**Table 2:** Oscillometric measures and CMR-derived parameters of the study cohort.

	**study population**
**Oscillometric measures**	
PWV_OSC_, m/s	6.7 ± 1.8
RR_sys_, mmHg	129 ± 14
RR_dia_, mmHg	85 ± 11
RR_sys_ central, mmHg	119 ± 14
RR_dia_ central, mmHg	86 ± 12
**CMR-derived measures**	
PWV_CMR_	6.1 ± 1.8
DC aA, 10^-3^mmHg^-1^	5.5 ± 3.3
DC dA, 10^-3^mmHg^-1^	5.3 ± 2.6
DC abA, 10^-3^mmHg^-1^	9.2 ± 3.5
DC mean, 10^-3^mmHg^-1^	6.7 ± 2.9
DC aA central, 10^-3^mmHg^-1^	7.7 ± 4.8
DC dA central, 10^-3^mmHg^-1^	7.3 ± 3.8
DC abA central, 10^-3^mmHg^-1^	12.6 ± 5.2
DC mean central, 10^-3^mmHg^-1^	9.2 ± 4.3
Aortic length R1, mm	121 ± 20
Aortic length R2, mm	169 ± 17
Aortic length R3, mm	290 ± 27

PWV_OSC_: pulse wave velocity assessed by transformation of oscillometrically defined brachial pressure wave form, RR_sys_: systolic blood pressure, RR_dia_: diastolic blood pressure, DC_aA_: distensibility coefficient of the ascending aorta, PWV_CMR_: pulse wave velocity assessed by cardiac magnetic resonance, DC_aA_: distensibility coefficient of the ascending aorta, DC_dA_: distensibility coefficient of the descending thoracic aorta, DC_abA_: distensibility coefficient of the abdominal aorta, DC mean: mean DC of all three aortic levels, DC central: DC at different aortic levels calculated using central aortic pulse pressure, R1: aortic arch, R2: descending thoracic to abdominal aorta, R3: R1 + R2 (Fig. 1b).

### Correlation of PWV with clinical characteristics and CMR parameters

PWV_OSC_ as well as PWV_CMR_ showed a very strong correlation with age (all r > 0.820, all p < 0.001). Correlation of brachial systolic (z-score: 1.484, p = 0.138) and diastolic (z-score: 0.869, p = 0.385) blood pressure and PWV did not differ significantly between PWV_OSC_ and PWV_CMR_ (table 3). The association between PWV_OSC_ and central systolic (z-score: 0.546, p = 0.585) and diastolic (z-score: 0.125, p = 0.900) blood pressure was not different from the association with peripheral blood pressure. Body mass index was moderately correlated with PWV_OSC_ and PWV_CMR_ (all r > 0.400, all p < 0.010).

**Table 3:** Linear correlation between PWV, clinical characteristics, oscillometric measures and CMR-derived parameters.

	**PWV_OSC_**		**PWV_CMR_**	
**Clinical characteristics**	**r**	**p**	**r**	**p**
Age, years	**0.878**	**<0.001**	**0.825**	**<0.001**
Body mass, kg	0.315	0.048	0.247	0.124
Height, cm	-0.056	0.733	-0.036	0.826
Body mass index, (kg/m^2^)	**0.549**	**<0.001**	**0.407**	**0.009**
Body surface area, m²	0.196	0.225	0.156	0.336
**Oscillometric measures**				
RR_sys_, mmHg	**0.683**	**<0.001**	**0.454**	**0.003**
RR_dia_, mmHg	**0.714**	**<0.001**	**0.600**	**<0.001**
RR_sys_ central, mmHg	**0.745**	**<0.001**	**0.531**	**<0.001**
RR_dia_ central, mmHg	**0.728**	**<0.001**	**0.610**	**<0.001**
PWV_OSC_, m/s			**0.859**	**<0.001**
**CMR-derived measures**				
DC_aA_, 10^-3^mmHg^-1^	**-0.783**	**<0.001**	**-0.627**	**<0.001**
DC_dA_, 10^-3^mmHg^-1^	**-0.751**	**<0.001**	**-0.619**	**<0.001**
DC_abA_, 10^-3^mmHg^-1^	**-0.654**	**<0.001**	**-0.502**	**0.001**
DC mean, 10^-3^mmHg^-1^	**-0.790**	**<0.001**	**-0.629**	**<0.001**
DC_aA_ central, 10^-3^mmHg^-1^	**-0.775**	**<0.001**	**-0.615**	**<0.001**
DC_dA_ central, 10^-3^mmHg^-1^	**-0.747**	**<0.001**	**-0.605**	**<0.001**
DC_abA_ central, 10^-3^mmHg^-1^	**-0.690**	**<0.001**	**-0.535**	**<0.001**
DC mean central, 10^-3^mmHg^-1^	**-0.784**	**<0.001**	**-0.621**	**<0.001**

PWV_OSC_: pulse wave velocity assessed by transformation of oscillometrically defined brachial pressure wave form, PWV_CMR_: pulse wave velocity assessed by cardiac magnetic resonance, RR_sys_: systolic blood pressure, RR_dia_: diastolic blood pressure, DC_aA_: distensibility coefficient of the ascending aorta, DC_dA_: distensibility coefficient of the descending thoracic aorta, DC_abA_: distensibility coefficient of the abdominal aorta, DC mean: mean DC of all three aortic levels, DC central: DC at different aortic levels calculated using central aortic pulse pressure.

Correlation coefficients between PWV and DC are summarized in table 3. There were no significant differences in linear correlation between PWV and DC of the ascending (z-score: -1.362, p = 0.173), descending thoracic (z-score: -1.083, p = 0.279) and abdominal aorta (z-score: -0.990, p = 0.322) when using either PWV_OSC_ or PWV_CMR_. Using central pulse pressure for the calculation of DC (DC central) resulted in similarly high correlation with PWV as DC derived from brachial pulse pressure (table 3). PWV_OSC_ rather than PWV_CMR_ showed a very strong association with mean DC of all three aortic levels (r < -0.790, p <0.001 vs. r < -0.629, p < 0.001).

### Comparison of PWV_OSC_ and PWV_CMR_

**Figure 2:**
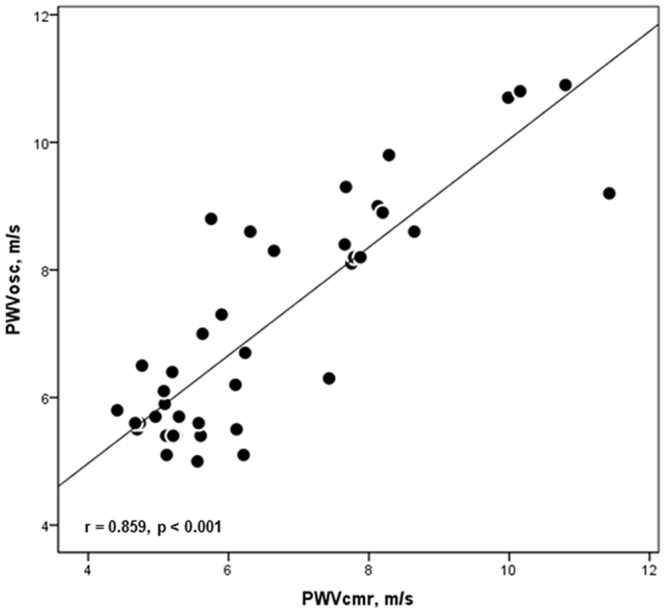
Correlation of aortic pulse wave velocity assessed by the two methods. Linear correlation between aortic pulse wave velocities assessed by brachial oscillometry (PWV_OSC_) and cardiac magnetic resonance (PWV_CMR_).

PWV_OSC_ showed a very strong correlation with PWV_CMR_ (r = 0.859, p < 0.001) (Fig. 2). There was a statistically significant difference in absolute values between PWV_OSC_ and PWV_CMR_ (p = < 0.001).

**Figure 3:**
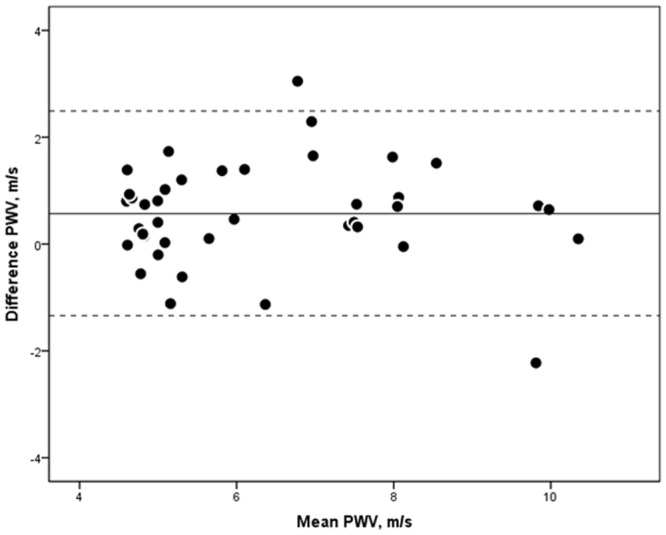
Methods´ agreement. Bland-Altman plots representing the agreement between the oscillometric method and cardiac magnetic resonance for the assessment of aortic pulse wave velocity. Corresponding coefficient of variation was 15%.

Bland-Altman plots of agreement between the methods are shown in Fig. 3. The bias was 0.57 m/s (SD = 0.96 m/s; upper and lower limit of agreement: 2.49 m/s and -1.34 m/s). The coefficient of variation was 15%, respectively.

## Discussion

Cardiac magnetic resonance imaging provides a valid and robust method for the assessment of aortic PWV [7, 20] but its application is limited in daily clinical practice and in acute illness. We raised the question if a simple and widely available oscillometric device could be a valid alternative for the assessment of PWV. The present study demonstrated (1) a strong correlation between PWV_OSC_ and PWV_CMR_ as well as (2) local parameters of aortic stiffness, (3) acceptable agreement between both methods and (4) higher PWV values if assessed by brachial oscillometry. These findings suggest the potential use of the new oscillometric approach as a non-invasive PWV assessment in a routine setting of clinical practice.

### Study population

We aimed to compare PWV_OSC_ and PWV_CMR_ in an unselected cohort of volunteers presenting without any known cardiac disease. At least one cardiovascular risk factor (hypertension, hyperlipidemia or smoking) was present in 7 subjects (18%). Mean PWV_CMR_ was 6.1 ± 1.8 m/s which is in good agreement with age-matched data from literature [21]. Males showed significantly higher PWV_OSC_ and PWV_CMR_ than females. According to a systematic review analysis, sex is not independently associated with carotid-femoral PWV [22]. No differences between males and females were observed for CMR-derived PWV [23]. Thus, higher PWV in males as shown in the present study might be due to a trend to higher age in the male group.

### Correlation of PWV with clinical characteristics and CMR parameters

We detected a very strong correlation between PWV and age. Independently of other risk factors, age is generally accepted as the main determinant of vascular stiffness [24–26]. Previously published data suggest a non-linear increase of PWV mainly occurring after the fifth decade of age [24]. Furthermore, our study confirms the well-established association between PWV and blood pressure [22, 27]. Remarkably, there was a trend to stronger correlation for PWV_OSC_, probably due to the fact that it is calculated by transformation of the brachial pressure wave form [8]. In line with our findings, Nunan et al. reported a close association of brachial cuff-derived PWV and age as well as systolic blood pressure, especially in participants older than 50 years of age and those with hypertension, as derived from a large community dwelling population [16]. The associations between PWV_OSC_, age and blood pressure were very similar to those obtained using non-invasive reference technologies, such as applanation tonometry [28].

Besides PWV, aortic distensibility is commonly used to characterize aortic function [5, 20]. We have previously shown that beside PWV_CMR_ assessment, calculation of distensibility coefficients is a robust method for assessing local aortic stiffness [19]. The strong correlation between PWV_OSC_ and aortic distensibility confirms the validity of the new oscillometric method for the assessment of aortic stiffness.

### Comparison of PWV_OSC_ and PWV_CMR_

In the present study, linear correlation between PWV_OSC_ and PWV_CMR_ was strong. However, there was a significant difference in absolute values between the two methods. A comparison between PWV_OSC_ and PWV derived from intra-aortic catheter measurements has been performed before by Hametner et al. [10]. Similar to the results of the present study, application of the oscillometric method resulted in higher PWV values [10]. These findings suggest a trend towards overestimation when assessing aortic PWV by transformation of brachial pressure wave forms. Presumably, this is due to the amplitude of the pressure wave as well as PWV being higher in peripheral arteries than in the aorta [2]. Hence, higher PWV_OSC_ values could be explained by the fact that the brachial pressure waveform is transformed for reconstruction of central hemodynamic patterns. Using a multiple-variable transformation process [9, 10, 29] instead of direct measures of aortic lengths might be another reason for higher PWV_OSC_ values.

Age-related elongation of the aorta is almost entirely due to the aortic arch, whereas distal aortic segments remain constant with age [23, 29]. Aortic lengths as measured in the present study (table 2) are in line with CMR-derived data from literature (aortic arch: 121 mm vs. 108 mm; ascending to abdominal aorta: 290 mm vs. 303 mm) [23].

Different PWV values when assessed by different methods might also be explained by regional changes in aortic stiffness. Hickson et al. previously reported a CMR-based study addressing age-related differences in regional aortic stiffness [23]. Lowest PWV was detected along the aortic arch and highest PWV in the abdominal aorta. Moreover, they detected higher values for total aortic PWV when assessed as carotid-femoral PWV using applanation tonometry compared to the CMR approach. The fact, that carotid-femoral PWV excludes the aortic arch but includes the low-elastic carotid, iliac and femoral arteries, might be responsible for this finding [23]. In the present study, PWV_CMR_ measurements excluded the most distal aortic segments towards the bifurcation. This might also explain lower values of PWV_CMR_ compared to PWV_OSC_, as detected in the current study.

Bland-Altman analysis of methods´ agreement showed a bias of 0.57 m/s (SD = 0.96 m/s) and the corresponding coefficient of variation was 15%. With regard to the ARTERY Society guidelines for validation of non-invasive devices to measure aortic PWV, brachial oscillometry fulfilled acceptable accuracy criteria compared to CMR in the present study [30]. For comparison, mean differences between invasively measured PWV and carotid-femoral PWV ranged between -0.2 m/s and -3.3 m/s, depending on the method to estimate the pulse wave travel distance [31].

### Applicability of PWV assessment using different modalities

Carotid-femoral PWV has been shown to be an independent predictor of all-cause mortality and cardiovascular morbidity in the general population [32] as well as in patients with cardiovascular and renal diseases [33, 34]. The use of carotid-femoral PWV is limited by the need of trained operators. Moreover, estimation of travel path length plays a pivotal role and is still discussed controversially [31, 35–37]. According to Weber et al., subtracting carotid—suprasternal notch distance from suprasternal notch—femoral distance provided best agreement with invasive PWV measurements [31, 37].

Invasive measurement of PWV is merely indicated in patients undergoing cardiac catheterization, what restricts this method to a highly specific patient group.

High cost and time efforts do not justify the use of CMR solely for PWV assessment. Nevertheless, PWV assessment can be easily embedded into a standard CMR-protocol [12]. Accordingly, CMR might provide an acceptable modality to assess aortic PWV in patients undergoing CMR imaging in clinical routine. Importantly, accuracy in the depiction of the aortic flow curve mainly depends on the temporal resolution of the CMR protocol. Higher temporal resolution would result in a longer acquisition time. According to Wentland et al. a CMR repetition time of approximately 10 ms, as used in the present study, allows for an accurate depiction of the aortic flow curve [38]. As shown in Fig. 1, the foot and systolic upstroke of the flow curve could be clearly delineated by means of the temporal resolution used in this study.

Brachial oscillometry provides a valid, safe, applicable and economic method to non-invasively assess aortic PWV [10, 15]. These facts suggest the use of brachial oscillometry for PWV assessment in the general population and several patient cohorts.

### Study limitations

There are some limitations that must be taken into consideration. Invasive assessment of central hemodynamics has not been performed in the present study. However, intra-arterial catheter measurements but not CMR are the generally accepted gold standard for the assessment of aortic PWV. This fact might be a major limitation when comparing PWV_CMR_ and the new oscillometric approach. Nevertheless, direct comparison of PWV_OSC_ and PWV_CMR_ with intra-aortic measurements, performed in previous studies, has shown good agreement [7, 10]. Moreover, according to the PWV validation guidelines, homogeneous gender distribution (at least 40% for either gender) as well as homogeneous distribution along age groups (<30, 30–60, >60 years) was recommended [30]. Of note, in the present study only 11 (28%) participants were female and despite showing a relatively wide age range, the median age of the cohort (34 years, IQR: 24–55 years) was rather low. Therefore, future studies are needed to confirm our results. Hemodynamic alterations might also interfere with brachial pulse wave analysis. Therefore, applicability and validity of PWV_OSC_ must be separately confirmed in the setting of different pathological conditions, prior to routine clinical use.

## Conclusions

Aortic PWV simply assessed by transformation of the oscillometrically-derived brachial pressure waveform shows an acceptable agreement with the CMR-derived transit time method according to the ARTERY Society guidelines. Nevertheless, absolute PWV values are higher when assessed by mathematical transformation of brachial pressure wave forms. Therefore, the two techniques should not be used interchangeably. Since decreased aortic elasticity is an independent predictor for cardiovascular morbidity and mortality, a non-invasive, economic and widely available tool could be quite useful for PWV assessment in daily clinical practice.
